# Negotiating Care: The Biographical Narratives of Young Adults Who Questioned Their Gender When Younger

**DOI:** 10.1111/1467-9566.13877

**Published:** 2024-12-28

**Authors:** Karl Atkin, Christine Jackson‐Taylor

**Affiliations:** ^1^ Department of Sociology University of York York UK

## Abstract

Current discussions about gender identity are increasingly politicised, particularly in the UK. An individual's body becomes a site of competing interests that attempt to regulate the physical, social and moral boundaries between biological sex and a socially realised gender. Care becomes defined within this context. The emerging biopolitics generates dividing practices that classify and regulate rather than situate a knowing subject, who is creatively and actively responsive. In response, our paper explores the value of biographical narratives when understanding how young adults negotiate their identity, within the context of social relationships and normative conventions, when experiencing (and articulating) gender questioning. Using testimonial experience from 18 young adults, aged between 19 and 30 years old, our analysis discusses participants' response to gender questioning before engaging with their exploration of gender, from childhood through to the present day. We consider how these young adults resolve gender questioning through reflexive engagement, seek legitimation and negotiate the response of others, as they seek care. Our conclusion, by providing insights into an actively constituted biographical experience, assesses the possibilities associated with more inclusive caring practices, in which an individual can flourish.

## 1 Introduction

Discussion of gender identity is increasingly politicised, especially in the UK, where a person's body becomes a site of competing interests that attempt to regulate the physical and moral boundaries between biological sex and a socially realised gender (Connell 2012). Attempts to ascribe the body create the potential for biographical narratives to assume an instrumental purpose, when negotiating care (Pearce 2018). The emerging biopolitics generates dividing practices that classify and regulate rather than situate a knowing subject, who is creatively and actively responsive (Jasanoff 2004). Public discourses, for example, can misrepresent experiences, as individuals struggle to find an ‘authentic’ voice, through which to articulate their identity (Zottola et al. 2023). Taking this as our starting point, our paper explores the negotiation of trans and gender affirming care among young adults. By enabling individuals to ‘think’ through their story (Frank 2000), we use testimonial experience to interrogate the potential for inclusive caring practices.

This experience can become lost as cultural and political debates about the rights (and lives) of trans and gender diverse people create a ‘stormy public discourse’ (Cass Review 2024). Controversies about suitable outcomes and models of care remain unresolved. Some, for example, regard current support as insufficiently explorative, by failing to adequately consider the circumstances in which a person questions their gender (Cass Review 2024), while others regard gender identity clinics as inappropriate gatekeepers that prevent individuals from realising their affirmed identity (Horton 2023). To enable a reimagining of current (and future) care, consistent with the intricacies of a person's life, our research explores the value of biographical narratives, in understanding how individuals make gender ‘work’ for them (Mol, Moser, and Pols 2010). By highlighting performativity, our analysis identifies the importance of trajectory, in which a continuing self‐reflection creates aspirations beyond the immediate. Young adults require a care able to respond to this, irrespective of the contingencies it creates.

## 2 Exploring Gender Identity

Care can evoke a positive and responsive nurturing, by prioritising ‘other‐centeredness’ (Engster 2005) and the ‘narrative turn’ by representing the social fabric, through which experience is articulated (Ricoeur 1991), offers a particularly valuable lens, to explore the positionality of gender identities beyond the cis‐normative (Prosser 1998). A conceptual uncoupling of social gender from biological sex opens up opportunities, which question the extent that gender is a ‘truly dichotomous variable’ (Epstein 2007, 236). Gender instead represents an emergent social experience, which is preformative, relational and dynamic (Butler 1990). Consequently, an alternative epistemic vocabulary able to provide expression to previously veiled voices becomes possible (Fricker 2007). These ‘disruptive’ voices can generate resistance (Mishler 2005), while creating an ethic of care that enables individuals to prioritise what matters to them (Singleton and Mee 2017).

Care, however, is never ‘disinterested’ but obliges participation (Martin, Myers, and Viseu 2015, 34). Narrative meaning is open to (re)interpretation, defined and understood through the predisposing processes of negotiation (Gilding 2010), in which strategic and personal agendas become actionable through social, political and cultural engagement (Tironi and Rodriguez‐Giralt 2017). This, while liberating, can inhibit (and cloud) possibilities (Fricker 2007). Our story, although individual, is located within an array of publicly circulating narratives. Testimonial experience, therefore, becomes part of a contentious discursive space, in which transformative potential vies with normative and regulated conventions, to circumscribe intent (Dewey and Gesbeck 2017). This impacts on how individuals understand and present their experiences and on how others perceive these experiences (Zottola et al. 2023).

Continually evolving diagnostic criteria, by facilitating access to treatments that enable an individual to legitimate and embody their gender identity, reflect this (Epstein 2021). Affirming technologies that reshape the body, for example, have to co‐opt a medical authority (Hilario 2017). This not only encourages an individual to locate their personal discomfort within a ‘dis‐ordered’ and medicalised body but also in relation to narratives that can gender their biographies in a particular way (Johnson 2019). To access treatments individuals may have to convince a clinician that they experience *gender incongruence*, by demonstrating a wish to live and be accepted as a member of the opposite sex and/or a need to make their body as congruent as possible with their desired sex (ICD 2024); or *gender dysphoria*, in which the individual exhibits a persistent cross‐gender identification and a discomfort with their assigned sex (DSM‐5 2023). Consequently, there remains a possibility that individuals, to be regarded as ‘authentically’ trans, become defined by a hegemonic binary, associated with masculinity and femininity (Garrison 2018). This maintains heteronormativity (where gender roles are naturalised according to a notional binary between men and women) and cisnormativity (where sex and gender are assumed to be linked in a constant way), while judging a person's trans‐ness (transnormativity) according to a ‘hierarchy of legitimacy’ (Johnson 2016, 466). A trans person is commonly represented as someone who identifies with a sex/gender opposite to the one assigned at birth, experiences dysphoria/incongruence and pursues an embodied transition, to enable them to pass in their assumed gender (Zimman 2014). This, however, can encourage a performativity, which may not be consistent with an individual's experience.

Gender, although negotiable, remains a situated and embodied experience (Epstein 2007). ‘Gender practice is powerfully shaped by the gender order around us’ and although we make our own gender, ‘we are not free to make it just as we like’ (Connell 2012, 874). Competing interests can, therefore, have a formative impact, to create a logic of care able to frame autonomy, irrespective of personal agency (Jeffreys 2014). Care is entangled with values, as it responds to vulnerability (Gill, Singleton, and Waterton 2017). This requires those who question their gender to reflect on how their voices are accorded credibility, when establishing a narrative, in which they can recognise themselves (Mishler 2005). By prioritising the specificity of these voices, our paper provides an understanding of ‘the creative calibration of elements’ (Mol 2006, 410–411), in which individuals negotiate their subjectivities within ascribing social contexts, to ‘get across the importance of good care’ (Mol, Moser, and Pols 2010, 2).

## 3 Doing the Research

Our research, commissioned by (but independent from) the Cass Review (https://cass.independent‐review.uk/research/), obtained Health Research Authority approval (IRAS project ID: 306023). Taking place between March 2022 and December 2023, it presents the experiences of young adults, who pursued gender‐affirming care. The study aimed to explore how both young people and young adults respond to gender questioning and negotiate its resolution by accessing formal support. To achieve this, we wished to prioritise a person's story (Riessman 2007), particularly as we were researching a contested topic, where misrepresentation is commonplace and at times, insufficiently responsive to trans and non‐binary testimonies. Our purposive sampling framework used multiple avenues of recruitment, to ensure we captured diversity in gender expression, including different approaches to transitioning. Six community and voluntary organisations who, in addition to engaging with valuable discussions about the research, circulated invitations to take part in the research. These organisations held a range of ‘positions’ on supporting those exploring their gender identity. We also sampled young adults using one of England's Adult Gender Identity Clinics and recruited a few, who asked to take part in research and met our sampling criteria. Participants came from across Great Britain. Recruitment, however, was not straightforward. Potential participants often commented on how the nature of public discourse can generate a sense of caution and anxiety, when engaging with research, which is increasingly distrusted.

When inviting people to take part in the research, we made no assumptions about how they may wish to identity. We interviewed 18 young adults aged 19–30 years old (see Table 1). Our sample included eight trans men and four trans women. Four young adults identified as non‐binary and/or gender fluid, including two who used to identify as trans but no longer used the label. Two participants described themselves as de‐transitioners. Five young adults were neurodiverse. Three identified as belonging to a racialised minority.

**TABLE 1:** Summary of sample characteristics, young adults.

Name	Participant self‐identification
Ali	A 30 year old trans femme/gender queer person.
Avery	A 19 year old, who does not identify with any gender, but once regarded themselves as trans.
Ben	A 19 year old trans man.
Blake	A 26 year old, trans woman.
Cameron	A 20 year old trans man.
Chloe	A 20 year old trans woman.
Daniel	A 26 year old trans man.
Dominic	A 28 year old trans man.
Freya	A 26 year old trans woman.
Hayden	A 26 year old trans non‐binary person.
Jack	A 29 year old trans man.
Jesse	A 22 year old who identified as trans when younger but no longer uses gender labels.
Joshua	A 27 year old trans man.
Liam	A 28 year old trans man.
Morgan	A 21‐year‐old, who is detransitioning.
Oliver	25 year old trans man.
Robin	A 28 year old trans man.
Sarah	A 30 year old who is detransitioning.

Interviews asked the young adults to reflect on their negotiation of gender, in addition to their experiences of care. While narrative in approach, in which we encouraged young adults to tell their story, we wanted to cover similar ground with our participants, to ensure we could compare responses, while creating an environment that enabled them to reflect on their specific experiences. A topic guide was developed from the relevant literature and through discussions with young gender diverse people as part of our patient and public involvement (PPI) work. They also commented on the make‐up of the sample and study focus. Interviews were held online using video conferencing software and lasted between 60 and 90 minutes. With consent, we audio recorded and transcribed interviews. We interviewed most participants once but adjusted our approach to support their needs. Neurodiverse participants, for example, were able to meet with a researcher for shorter periods and—if requested—we provided discussion topics beforehand. When analysing our material, we used open‐coding before generating themes by reviewing and exploring the significance of our codes for each interview. We then compared these themes, across interviews to highlight potential similarities and differences. We reflected on how these themes shaped interpretative meaning, by locating biographical experience, in which participants negotiate and ascribe meaning, within normative expressions of gender identity. This enabled us to relate personal (and cultural) stories to social context (Gubrium and Holstein 2009).

## 4 Negotiating Gender Identity

Our analysis engages with how young adults reconcile interpretative meaning with an active and embodied agency, to generate a knowing social presence, enfolding in time. This, by prioritising an individual's responsiveness to changing environments, as they attempt to align a positive self‐identification with social expectations, generated three interlinking themes, with which to interrogate caring possibilities. Our first theme explores how young adults respond to their gender questioning and seek resolution. Meaning and purpose is created through this spatial (and embodied) engagement, as is trajectory. There is uncertainty but also a sense of purposive (and creative) intent as young adults seek legitimation. This creates expectations about care and is the focus of our second theme. The potential for ‘other‐centeredness’ is established, as self‐reflection creates aspirations, which young people look to affirm and embody. When doing this, however, young adults negotiate alternative perspectives about what care (and trans identity) ‘should’ look like. Our third theme considers the vulnerability this generates, by exploring the situated nature of care, in which young people engage with the narratives that others may regard as a more suitable reference point, from which to provide care.

### 4.1 Making Sense of Gender Questioning

Creating an understanding, to access care, requires individuals to negotiate an emergent identity, in which uncertainty occurs alongside transformative possibilities. This informs young adults' search for resolution. Consistent with previous accounts (Zottola et al. 2023) young adults talked about a sense of difference, when growing up, but one they found difficult to ‘name’. Oliver, a trans man, explained:

I think in the back of my head I always was (trans). I was just never equipped with the language to describe it.

Gender norms confused young adults and made them feel uncomfortable. Relationships, they said, reinforced this discomfort. Young adults described how social interaction could be fraught with anxiety. Many said it was made clear to them when their behaviour did not meet others' expectations. They described bullying, hostility, dismissal and rejection. Jack was reluctant to disclose his trans identity because of this. A decision he now regrets:

I just wish I’d come out sooner, really. I did it when I was ready. I wish I could have been ready earlier because yes, I lost a lot of time.

Reimagining a conventional relationship between biological sex and social gender offered—for some—a potential solution. Many young adults said they did not seek—nor were encouraged—to disrupt gender stereotypes by recreating them as a (fluid) continuum; at least initially.

This created a vulnerability, which finds expression in an initial sense of urgency, much of which, young adults explained, is focused on accessing medical pathways, to ‘fix the problem’, consistent with a gender binary. This, they said, gave certainty and offered resolution. Their priority was to pass—and be accepted—in their affirmed gender. Over time, young adults re‐negotiated this urgency. For some, this was because they had no choice. Avery was unaware that their mother had declined a referral to specialist gender identity service. Left without support, while a teenager, they felt their life could not begin unless they accessed gender‐affirming care:

From aged 13 to aged 17, yes (…) I wanted to take testosterone, I wanted to have surgery. I was completely, yes, no doubt in my mind that that was what I wanted (…) I identified as male and I wanted people to perceive me the same way that I felt about myself inside. So, I just thought that medical transition was the right decision.

Joshua explained how repeated delays to surgery had prevented them from leading the life he wanted: ‘Because even for me now, it's like I don't feel my life can properly start’. Joshua had to seek alternative ways to express his gender. For others, the pursuit of emergent and more fluid opportunities enabled them to challenge cisnormativity. Access to life‐affirming medical pathways, to realise these opportunities required, however, engagement with a diagnosis.

When negotiating access to care, young adults felt they had to negotiate a diagnosis, which encouraged them to associate their feelings of difference with ‘dysphoria’ and/or ‘incongruence’. Many young adults recognise that ‘dysphoria’ can be a useful diagnostic category as it provides an opportunity to legitimise their experience and justify asking for transformative care to enable them to affirm their gender. Young adults also remarked that diagnosis created a possible shared language, which others could understand, as a counter to the hostility they experienced. Diagnosis, therefore, had strategic and instrumental worth, although this is not without ambivalence (also see Johnson 2019). Inherent to this ambivalence, was a belief that the diagnostic process had discouraged them to think beyond a gender binary. Performativity, according to a learnt script, was prioritised, in which young adults used a language, which was not entirely their own, to gain access to life‐affirming treatments and social acceptance. Young adults maintained the value of diagnosis, as a means of facilitating access to medical pathways, alongside an appreciation that it could be an unhelpful medicalisation. Some, for example, expressed concerns that a social experience could only be regarded as legitimate if a medical label were attached. A few expressed ‘anger’ about what they regarded as inappropriate gatekeeping, which created unnecessary barriers that prevented them from affirming their identity.

Looking back, young adults became aware that a diagnosis did not define them, in the way they believed it did, when younger. Most accepted diagnosis as a necessary process, but one that necessitated a ‘credibility check’ on what an ‘authentic’ trans identity meant (Hilario 2017, 558). Resistance was, therefore, required as young adults negotiated their own vulnerabilities, relative to a diagnostic essentialism that allowed little opportunity for further exploration. They explained how an emerging understanding of diverse LGBTQIA identities and communities enabled greater self‐awareness. Young adults sought experiences consistent with how they wanted to live their gender. Several describe ‘relief’, when discovering that they were not alone. A few, however, recognised that much depended on the diversity available, including access to identities beyond the binary. Understandably, they remain sensitive to possible accusations that they only begin gender questioning, once discovering different expressions of gender. Gender questioning, they said, came first and ‘naming’ did not create them. For many, the discovery of gender diversity—and the potential associated with different ways of transitioning—brought comfort.

Oliver reflected on the importance of taking things at your own pace and although he felt others' stories had a value, it was important not to use them, to compare and judge your own experiences:

And it wasn’t kind of this massive catalytic breakdown that sometimes you see portrayed in the media or that you hear a lot of trans people talking about, it was peace and understanding of “Oh this is why I don’t feel great about myself, this is why I don’t like my body, this is why I struggle with a lot of things.”

Many young adults remarked that the diversity of transition was not initially apparent to them. Seeking alternative accounts, which opened up more fluid identifications and expressions, gave young adults confidence, when challenging more rigid accounts of gender conformity. Ben, a trans man, explained that they began to experiment with pronouns online and then began a social transition, first at home and then with friends. This created space that enabled him to reflect on what he wanted:

I guess in a way I was quite content with how I was. I was, like, that was enough for then, like changing my name and my pronouns was enough for me at that point (…) So, to be honest I didn’t know really that surgery or hormones was an option, but I think even if I did I don’t think I would have proactively done anything because even when I did find out I was, like, I’m okay as I am right now, so there wasn’t any attempt to find anything else where I was quite content in just friendship circles.

As Ben got older, his relationship with his body shifted. Puberty increased his anxiety and discomfort. Ben discussed medical interventions with his parents, who felt he was too young to access medical interventions, although when 18 Ben used testosterone to support his social transition:

Yes, I think that’s to do with me growing up and to do with maturing as an individual as well. It’s, like, a mix of things of, like, as I grow up I get more educated, I get the right vocabulary, I’m able to have the confidence to advocate for myself more. There is also a bigger community that I’ve connected with so I’m more knowledgeable as an individual

Ben's observations about having the ‘right vocabulary’, which he developed through exploration, is telling, as is the use of this vocabulary, as the basis for affirming his transition.

With hindsight, young adults realised they had more flexibility (and time), than they first realised. Gender identity evolved as young adults searched for an identification, with which they felt comfortable. Self‐discovery brought ‘happiness’ and ‘joy’, as young adults develop an affirming and anticipatory understanding, by creating a space to live a gender, congruous with how they feel. Euphoria featured in many narratives, along with hope. Hayden, who is trans non‐binary explained: ‘I couldn't actually relate loads to the binary trans experiences’, when experiencing an emergent ‘euphoria’:

I’ve been out for about five (years) now, so the past two years have been really wonderful in the sense that I felt confident, I’ve felt like I have the right to take up space and I have the right to just be me and that’s where I started going, oh so what does your euphoria actually want? What are the things that make you happy? And exploring my gender in that way has been just such a joy.

Hayden no longer focused on what they felt was lacking in their life:

The messages that we consume, they are very much like: what are you not? Rather than what you can be and what you are, what would bring you joy rather, save yourself from this uncomfortable feeling.

Developing confidence became especially important to them:

But it really just had to come with the experience of living as a non‐binary person to then have the confidence to explore it in a positive way.

Hayden's point, about the importance of rejecting a performativity, premised on ‘what you are not’, found echoes in the narratives of other young adults. This enabled gender to become a positive journey of celebratory and empowering discovery. Young adults could now define who they are, on their own terms, rather than how others may see them, and although never entirely liberated from this gaze, their narratives can be used to challenge what is expected of them. For many this is entirely consistent with being trans, of which they are proud. For others, it is associated with non‐binary and/or gender queer expressions. Some find comfort in reinventing a more flexible gender binary. Others wish to break free of it.

### 4.2 Affirming and Embodying Gender Identity

Young adults expect their lived gender identity to be respected, although many remain open to exploring what this means for them and their body. They do not regard this as contradictory but an act of agency that enables them to pursue opportunities, consistent with their values. Understanding that they do not have to conform to attempts by others to gender their experience generates confidence. They are clear, gender identity is not a lifestyle choice (or preference) nor is it an ‘adaptable’ identity that can be disregarded from one day‐to‐the‐next. Its social or material expression, however, is flexible. Care, young adults say, is required to recognise this. Ben remarked:

Because not everybody, kind of, falls under this neat little umbrella (…). Some people go halfway, some people don’t go all the way, some people just need advice, you know (…) much like gender, it’s not confined to little boxes (…) because it will then exclude so many more people.

Ben, like many other young adults, argued for an inclusive care that is open and responsive rather than having a single reference point, with which to judge trans and gender questioning.

Young adults explain how their understanding can shift over time. They struggle with narratives that insist linear transformation/trajectory is required to render gender questioning intelligible. Several said that once you begin questioning your gender, you are continually aware of the possibilities associated with it. For many, gender expression became open‐ended, in which they accord value to (iteratively) ‘figuring out’ how to live in their body. Daniel, a trans man, explained that social transition, supported by medical pathways, enabled self‐awareness. This does not, however, start or end with a medical intervention:

But I think as a young trans person you’re under the illusion that actually you will just change, you know, you have these surgeries and that’s it, done, you’re a completely different person, but actually I’ve learnt that you grow into it and you start to understand yourself more and you begin to love yourself because of it and you appreciate what you’ve been through and all the trauma and everything that’s happened. It’s been a really sort of ‐ It’s been nice, it’s been really nice to have gone through that and understand that about myself and, although it’s been very, very difficult and very stressful, not just for me but also for my mum and my family, it’s made us closer and it’s done more than I could ever have imagined it doing actually, it’s had a much better outcome than I thought it would.

For Daniel, care requires sensitivity to emerging circumstances. He regards respect for the process of self‐realisation—and the ‘trauma’ it can create for the individual and their family—as a particularly important consideration, when establishing ‘good care’. Other young adults would agree.

As part of this self‐realisation, there remains flexibility in how young adults decide to embody their gender identity. This again challenges fixed accounts of performativity, associated with cisnormativity. Fifteen participants accessed medical pathways, including gender affirming hormones and/or ‘top surgery’ or ‘chest augmentation’. Of this group, four had phalloplasty or vaginoplasty. Several young adults remained on a waiting list for initial or subsequent phases of surgical intervention. Two had initially sought service referrals with the intention of accessing medical pathways but decided this was not right for them. One participant was not currently considering a medical pathway. Our account is not intended to reduce our participants to the sum of their transitions but to introduce it, as a way of providing an understanding of the complex ways in which young adults learn to embody their gender. Cameron, a trans man, who had accessed puberty blockers and testosterone, for example, felt this was a sufficient transition. He explained their position on ‘lower surgery’:

If it ain’t broke, don’t fix it (…) it’s the sense of, well, why would I need it? Like what is it going to fix? Like currently, I have a boyfriend and we’re looking to be pretty long‐term, so I don’t think my boyfriend has any issues with it, and like so far I don’t have any issues with it, and it doesn’t affect my personal life. It doesn’t affect my sex life. It doesn’t affect my day to day life, so it’s not important, you know.

Freya, a trans woman, explained how a prescription for oestrogen meant she no longer considered breast augmentation, although as she grew older, underwent vaginoplasty:

It got to the point where I think I felt that oestrogen had done its work and I felt my body had developed fully and I think a little bit of dysphoria was kicking in again. I was socialising during the summer and people were ‘let’s go swimming’ and I’m sort of like I’ll go swimming but I’ll be wearing shorts and things like that. I think that’s where I started to feel I had to go further with where I was and I think I felt ready for it.

Young adults believe that there is no one way of being ‘trans’, although they comment on the lack of discursive space that enables them to express this. Care, they feel, is positioned as facilitating a fixed (and one‐off) change from one gender to another, consistent with transnormativity (also see Johnson 2016). Several young adults observed that medical interventions are compatible with non‐binary or gender fluid expressions of identity, rather than exclusive transgender identities, although they feel that care does not always allow for this. For some, social transition is sufficient in expressing who they are. Others believe physical transition is required, although this can take different forms and be used in combination with social transition, which is neither fixed nor permanent. A few, however, encounter a more difficult journey, which further reinforces the importance of understanding reflexive agency, when negotiating a caring space that empowers them to first, understand their experiences and second, make the choices they feel are required of them. Avery, identified as trans between the ages of 13 and 17 and had socially transitioned, in some aspects of their life. They initially hoped to physically transition. Avery was bullied at school. An experience they describe as traumatic. They were also autistic and described difficulties with their mental health. They had been unable to access formal care, because of their parents' reluctance. Their gender identity shifted in ways that were embodied but also socially realised:

I remember, like the distinct point at which I realised that I’d made the wrong decision was lying in my bed at night and thinking about having a deep voice and passing as a male. And I was like, it didn’t make me feel any happier than I felt at the time. In fact, it made me feel a bit like I was trapped in a box, I feel like that’s the best way I can describe it. I feel the same way about the label of being female, I don’t want to have to fit in a box, but equally I don’t want to take hormones to not fit into a box. Because I feel like that’s just transferring boxes.

This necessitated a social transition, beyond their identity as a male or as female. They do not regard themselves as a detransitioner, despite finding the narratives of those who had detransitioned helpful. Avery, however, found gender labels less important, as they grew older.

Two young adults, however, did regard themselves as detransitioners. They pursued medical pathways, believing they would have a positive impact. For a short while, they did. Morgan explained that following a diagnosis of dysphoria, she decided to transition. After registering with a private provider (and having sufficient resources to do so, which was not available to all participants), she was referred for hormone interventions after a one‐hour appointment:

I thought, “Oh my god, I’m going to be able to go on testosterone soon, like in a few months.” I felt just a huge relief, the biggest relief that I’ve ever felt in my life, to be honest. And yeah, but at the back of my mind, I thought, “He (doctor) didn’t ask me about these things in my head.” And I thought, “Well, it doesn’t matter. He knows what he’s doing.” I trusted him that he was doing the right thing. But in the back of my mind, I thought, “But I have these other things that he never asked me about.”

Morgan had experienced ‘sexual abuse and neglect’ when younger. On reflection, she believed this was related to her gender questioning. Sarah followed a similar privately funded physical transition. She also spoke about a lack of opportunity to talk, although at the time this suited her

I was reticent (…) because I knew that if (clinician) got any kind of whiff of mental health problems that it would delay my treatment. So, I airbrushed my experiences in school, I airbrushed my depression, certainly mentioned nothing about suicide or self‐harm.

Sarah justified this, believing transition would help her find happiness:

As far as I was concerned I didn’t have any mental health problems because the mental health problems I did have were all linked to me being a closeted transsexual.

Their account reflected a great deal of internalised anger, particularly as she attempted to reconcile previous decisions, with who she was. Sarah had regarded herself as trans, although her current ‘anger’ meant she now interpreted her previous actions as ‘deceit’. Transitioning had not improved her life in the ways she had hoped, although it took her some time to realise this. Homophobic bullying at school, Sarah said, made it difficult for her to acknowledge her sexuality, which she confused with gender questioning. Following a recent emotional crisis—and with support of her long‐term partner—she now understood herself as gay. This was liberating but Sarah remained angry about how she had altered her body:

I now look, I look at my body in the mirror and I feel I have mutilated it, I have butchered it. I am in the process of trying to come to peace and accept that I was very unwell, I feel I was failed by a system that was by its design, unable to properly deal with someone like me, who was really suffering mental health problems and was not transsexual, and come to peace with the permanent changes that I have done that I have been allowed to do.

Sarah and Morgan believe there is little public space available, to explore experiences of de‐transitioning. Morgan remarked that ‘detransition was a bit stigmatised’:

I felt like it wasn’t, you know, acceptable to go back. It wasn’t a thing to go back, you know. It wasn’t something that was talked about. It didn’t feel like an option that they wanted to discuss or even mention (…) I want detransition to be something that can be openly talked about, and regret to be openly talked about (…) I think that it’s important for people to know that it might not be right for them. It might be, it might not be, and just kind of bear that in mind.

What is striking about the accounts of Morgan and Sarah is that although they position de‐transitioning as a distinct experience (and identity), their characterisation of care resonates with the narratives of the other young adults we spoke to. Morgan and Sarah talk about the creation of a space for unhurried exploration, in which an individual does not have to anticipate cis or transnormativity, to resolve embodied distress. Morgan, however, did not want her experiences to be used to undermine trans identities and deny life‐affirming pathways. Avery agreed and spoke about the importance of respect for diverse experiences:

I think that hearing from people who are trans, who have medically transitioned is great. Hearing from people who are trans who have chosen not to, hearing from people who have detransitioned is great too. I think that people often don’t want to present de‐transitioners’ perspectives because they feel that it’s I guess questioning the validity of trans people. But I think that de‐transitioners’ perspectives are really, really important because they can explain, I guess, the way that you might feel.

### 4.3 Negotiating Vulnerability

Expression, while fluid, does not, young adults argue, undermine an authentic underpinning identity, when establishing a meaningful lived gender. For young adults, ‘good care’ rested upon a space that enabled exploration of the social and material expression of gender, which respected trajectory, as an expression of agency, without doubting them as an authority on their experiences. This, however, created a precarity (and vulnerability), in which young people feel they have to justify their questioning, while encountering the possibility that their experience may not be believed. Most spoke of how their transition, irrespective of how it became embodied, enabled them to flourish. As Daniel, a trans man, who had physically transitioned through surgery, remarked, it was ‘absolutely the right thing’ for him to do, irrespective of the ‘trauma’ experienced, in which he had to challenge the continual scepticism that others associated with his experiences. Medical pathways are right for some people but young adults emphasise the importance of knowing you are no less trans, no less queer, no less non‐binary if you follow a more social transition. Ali explained the implications of this for their expectations of care and the importance of not acting out predetermined scripts, inconsistent with how you feel:

Identity is so rarely static, and I think, as a young person (…) when you’re questioning identity but not necessarily sure what it is, you can easily latch onto the first thing that feels right for you. For some people that might be the exact right thing but for other people, it might be something different and almost if there was permission to be able to explore without feeling like you’re being an imposter (…) If that was possible, I feel like that would lead to a more fulfilling gender exploration for questioning young people.

‘Permission’ to explore, without feeling ‘an imposter’ characterised other young adults' narratives. Many young adults come to understand that transition does not have to be linear or uniform. For them, trans is an inclusive identity. Young adults are keen to emphasise that individuals make different journeys, when expressing and legitimating their gender. Using someone's personal experience to undermine or deny that of another is regarded as a threat, in which narratives become ‘weaponised’ (see Táíwò 2022). Young adults bemoan the lack of public space that endorses the legitimacy of different journeys. Transitioning can bring a powerful sense of comfort and euphoria but as young adults remark, this has to be realised against the backdrop of hostility and discrimination, in which their experience is distrusted. Hayden explained how they felt continually judged by those from whom they sought care, which they regard as ‘harmful’, especially in how cisnormativity can define trans identities:

It’s also very cis centred, it’s like: if this cis person thinks that you’re trans enough you can go do this and all of the (…) I was like this is frankly horrible.

Young adults believe that opening up an acceptance for a diversity of transition that does continually require them to prove they are ‘trans enough’, is an important aspect of ‘good care’. That such a space is not available worries them, particularly as it can prevent individuals from exploring options that are right for them. Liam, a trans man, explained the importance of this:

Piece together why I was feeling the way that I was. Yes. It was difficult, at times, I suppose but I’m glad I had to go through it.

The possibility of internalised and socially realised transphobia, homophobia and misogyny concerned young adults, as did reinforcement of hetero and cisnormativity. Blake, for example, said they were beginning to ask some difficult and searching questions about their transition, which they correlated with increasingly negative public discussion, particularly about trans women. This meant they had to ‘work through dark stages’:

I think it very much depends on where this really heated discussion about trans people and society (…) goes. Because my self‐perception is directly ‐ and it’s terrible and I know I need to get a healthier sense of thinking about myself and how I frame my life and who I am but at the minute ‐ and actually always it’s been very much tied to the current discussion around trans people, whatever that is at the time. You know, when I was younger it was very much “Oh yeah, this is amazing, women can become men and men can become women” and there wasn’t really a lot of questions asked (…) you know, there’s part of me that really sort of misses that time in my life when the discussion was very sort of pro trans.

Here Blake vividly highlights the ‘colonisation’ of care by hostility to trans (and non‐binary) experiences, alongside the complexities of creating spaces for gender exploration. Blake—like other young adults—recognises that choices are continually required of them and that expectations associated with performativity can, at times, compromise these choices. Hostility, in which the credibility of narratives is doubted, further explains the value young adults place on affirmation, which they regard as non‐negotiable. This, however, does not preclude exploration, despite the risk that this can be associated with a ‘chaos’ narrative (Frank 2000). For young adults, choices require reinterpretation, as consequences cannot be foreseen and in which inaction can be as much a risk as action. Their experiences are, however, consistent with others, required to negotiate potential life‐affirming treatments (see McQueen 2017). For young adults this requires care, which respects the potential associated with their identity (including access to medical pathways), while not denying the possibilities of ongoing reflection, as they seek comfort, when understanding who they are.

## 5 Discussion

A ‘new gender paradox’ requires us to: ‘persist with the gender binary, which produces gender inequality, in order to fight it, and at the same time, fragment it more and more, so that it disappears’ (Lorber 2022, 82). Biographical narratives, by connecting body, identity and society, offer insights into the epistemic potential of this paradox, through which a reimagining of care is possible. The support required by those who question their gender identity is no different from anyone seeking care, as it reflects ‘persistent tinkering in a world full of complex, ambivalent and shifting tensions’ (Mol, Moser, and Pols 2010, 14). Young adults, when responding to trans and gender questioning, generate aspirations, which require negotiation. These are not always linear nor fixed but require them to make gender ‘work’. Care is sought on this basis, in which trajectories and their transformative possibilities vie with precarity and vulnerability to define care. Trans is ‘always narrative work, a transformation of body that requires the remoulding of life into particular narrative shapes’ (Prosser 1998, 4). The richness of our participants' account confirmed this.

Young adults require timely access to appropriate care, in which they are able to make decisions about their future. This includes safe and effective treatments, alongside respectful care, sensitive to the choices required of them. For young adults, ‘good care’ provides an iterative space, consistent with the contingent intricacies of their life. Situations and relationships enact, shape and reproduce care, as expectations—and their potential outcomes—become subject to (re)negotiation. Healing potential is, however, undermined by ideological inscriptions, which by claiming authority over the body, have the potential to generate trauma and subvert caring intent (Táíwò 2022). As young adults understand, care mediates their capacity to flourish. Their concern is to establish an ontology of trans, which can be recognised as legitimate by others, although in doing so, they believe they are judged and held accountable for their difference. Young adults feel especially vulnerable to accusations that they do not take their gender seriously or else, through an act of preference—or by adapting a particular lifestyle—have ‘chosen’ to be trans. Young adults are equally aware that they have to reconcile their experience against a social expectation, offering little opportunity for doubt and in which, a clear linear trajectory, associated with facilitating a fixed and permanent gender identity, becomes incompatible with ongoing exploration. Whether they are negotiating medical pathways or the doubts of others, there are occasions when young adults feel that they have no alternative but to conform to dichotomising discourses, even when they know that these may be inconsistent with how they feel. The legal and regulatory framework, with which they engage, such as the Gender Recognition Act—and the diagnostic process—reproduce and reinforce these expectations (Grabham 2010).

Cisgendered and heteronormative assumptions, therefore, can conspire with transnormativity, to hinder the development of an inclusive caring space and discourage ongoing reflection, consistent with what matters to them (Dewey 2013). Young adults agree that their initial response to gender questioning is an attempt to negotiate such assumptions. With experience, many come to live less rigid expressions of gender and gain confidence, when challenging gender binaries (also see Zottola et al. 2023), although tensions remain as young adults' narratives become continually situated and understood, within public narratives of (mis)understanding (Somers and Gibson 1994). Young people reinterpret their experience and reflect on the decisions they make, although they are reluctant to articulate this, especially as decision making is scrutinised, beyond the individual to offer a public commentary on care. Decisions, taken at particular time points generate consequences (McQueen 2017). This is not unique to gender questioning and occurs in other life‐changing transformative interventions, from reproductive technologies to cancer treatments (McKinnon 2015). Nonetheless, for those negotiating gender identity, exploration can be positioned as a reason for care to be discouraged or withheld. Reconciling the immediate, with a requirement to see beyond it, is what characterised the narratives of young adults. This necessitates a care that listens (Mol 2006). For young adults, gender is a lifetime's work, although as a requirement of listening, an important distinction is made between affirming an identity, which they regard as non‐negotiable and accessing a care that empowers an exploration of what this identity may mean for them and their bodies. This requires a particular temporality of care, which is open, uncommitted and non‐judgemental and realised through a willingness to continually respond, as situations change and unfold (Mol, Moser, and Pols 2010).

Assumptions about the relationship between biological bodies and social gender, however, generate opportunities for contestation (Singer 2015). Attempts to affirm a ‘euphoric’ identity can occur alongside a potential silencing strategy. Do young adults identify with an experience, inconsistent with how they feel, to assert a ‘frozen’ trans identity, likely to acquire some degree of social acceptance, amenable to care, but which requires them ‘to perform (and produce) a gender permanence in a way that non‐trans citizens are not required to do’ (Grabham 2010, 109)? Alternatively, do they persist with ‘tinkering’ (Mol, Moser, and Pols 2010, 14), in which non‐linearity and multiplicity, while empowering, is not accorded the same value as binary gender performance? Young adults told us about what matters to them: recognising the diversity of experiences that accompany gender questioning; acknowledging the different ways that gender identity finds expression and how this may change with self‐reflection. Nonetheless, they remain sensitive to how their narratives, by blurring boundaries, can be regarded as untrustworthy, inauthentic and at times, undesirable. Consequently, any transformative potential may be regarded as contingent and precarious; realised against a gender that maintains a moral and social order by remaining a ‘binary institution’ (Lorber 2022, 69). Young adults require a receptive audience, with the necessary epistemic competence, to understand their testimony (Dotson 2012), irrespective of the complexities generated and independent of an assumed performativity, which genders their biographies in a particular way, with little respect for trajectory. A more ‘trustful’ conversation is, therefore, necessary (Fricker 2007).

Social divisions risk the loss of care (Gill, Singleton, and Waterton 2017). Controversies and disagreements about appropriate models of support, diagnostic categories and suitable outcomes, along with essentialised and fixed expressions of gender identity, with no regard for trajectory, can threaten and subvert the ‘other centeredness’ of care. Considerable personal identity work is required by young adults to avoid the erasure of experiences that exist ‘between and beyond the binary framework’ (Darwin 2020, 361). Open engagement, by locating a knowing subject, who is creatively and adaptably responsive, relative to context, can enable a critical reimagining of care practices. A disobedience can help generate an inclusivity (Mignolo 2010), respectful of difference, in which a curious reflection, which can never be satisfied (Mol, Moser, and Pols 2010) generates an ethos of care in which any outcome ‘is (…) always until further notice’ (Bauman 1994, 200). This enables young adults to challenge the ‘hardening of the categories of the everyday’ (Singleton and Mee 2017, 133) and establish a care, regarded as a ‘wager on the unfinished nature of the present (…) not by predicting the future, but on the ability to lure events in the direction of new possibilities’ (Greco 2017, 122). A concern with the linear, in which a goal is established, treatment occurs and a fixed outcome recorded, is counter to how young adults experience the unfolding of care.

Contingency, however, should not be regarded as inconsistent with ‘good care’ (Singleton and Mee 2017). Instead, care should be empowered to unravel and articulate tensions, ‘by cast(ing) them in the words that may allow them to travel, so that they may be more widely reflected upon’ (Mol 2006, 211). When considering the hostile context in which gender questioning occurs, any ethos of responsive curiosity should not, however, position young people as confused or lacking capacity to act in their own best interests. This curiosity does not discredit or devalue those who feel certain about their gender identity—and wish to affirm its transformative potential through transition—nor should it exclude the voices of those who wish to re‐explore its meaning. Most of those we spoke regard themselves as positively trans and this is an important consideration, when interpreting their narratives. Care is required to support this, while allowing for iterative reflections, as part of a trans ontology, which includes space for those who have doubts. Polarising discourses, when representing collective identities, sensitive to multiple subjectivities (Hill Collins and Bilge 2016) can present others' independence as a threat (Carbado 2013). In opposing this, encountering the ‘other’, rather than an opportunity to exercise exclusionary and dividing practices, can instead be used to establish plural and creative modes of engagement that celebrate reciprocity and mutuality. As Fanon (1961) observed, the only subject is a living one and care is required to write this living into time. We risk (re)producing injustice if gender identity services do not empower this.

## Author Contributions

**Karl Atkin:** conceptualisation (equal), data curation (equal), formal analysis (equal), funding acquisition (lead), investigation (equal), methodology (equal), project administration (lead), supervision (lead), writing–original draft (equal), writing–review & editing (lead). **Christine Jackson‐Taylor:** data curation (equal), formal analysis (equal), investigation (equal), methodology (equal), project administration (supporting), writing–original draft (equal), writing–review & editing (supporting).

## Ethics Statement

The research received ethical approval from the Health Research Authority (IRAS project ID: 306023).

## Consent

Informed written consent was obtained from all participants.

## Conflicts of Interest

The authors declare no conflicts of interest.

## Data Availability

The interview material is not currently available because of the sensitivity of what was talked about and the high risk that participants could be identified. The study gained ethical approval on this basis.
